# Capsular polysaccharide enables *Klebsiella pneumoniae* to evade phagocytosis by blocking host-bacteria interactions

**DOI:** 10.1128/mbio.03838-24

**Published:** 2025-02-14

**Authors:** Xiaoxuan Liu, Qi Xu, Xuemei Yang, Heng Heng, Chen Yang, Guan Yang, Mingxiu Peng, Edward Wai-Chi Chan, Sheng Chen

**Affiliations:** 1 Department of Infectious Diseases and Public Health, Jockey Club College of Veterinary Medicine and Life Sciences, City University of Hong Kong, Kowloon, Hong Kong; 2 Department of Food Science and Nutrition, Faculty of Science, The Hong Kong Polytechnic University, Hung Hom, Hong Kong; 3 The State Key Laboratory of Chemical Biology and Drug Discovery, Faculty of Science, The Hong Kong Polytechnic University, Hung Hom, Hong Kong; 4 Shenzhen Key Laboratory for Food Biological Safety Control, The Hong Kong Polytechnic University Shenzhen Research Institute 597660 , Shenzhen, China; Iowa State University, Ames, Iowa, USA

**Keywords:** *Klebsiella pneumoniae*, virulence mechanisms, capsular polysaccharide, host recognition, host response

## Abstract

Capsule polysaccharide (CPS) is among the most important virulence factors of *Klebsiella pneumoniae*. Previous studies demonstrated that CPS plays multiple functional roles, but the mechanism by which this virulence factor enhances the survival fitness of *K. pneumoniae* remains unclear. In this work, we demonstrate that CPS is the main cellular component that not only elicits the host immune response to *K. pneumoniae* but also enables this pathogen to survive for a prolonged period under adverse environmental conditions. Consistently, our *in vitro* experiments suggest that CPS prevents *K. pneumoniae* from phagocytosis, rendering the encapsulated strain more difficult to be eradicated by the host. We also found that phagocytosis of *K. pneumoniae* is partially mediated by LOX-1, a scavenger receptor of the host, and that CPS may impede interaction between LOX-1 and this pathogenic bacteria, therefore reducing the phagocytosis process. These findings provide insights into the pathogenic mechanisms of this important clinical pathogen and should facilitate the design of new strategies to combat *K. pneumoniae* infections.

**IMPORTANCE:**

*Klebsiella pneumoniae* has become one of the most important clinical bacterial pathogens due to its evolution into hyperresistant and hypervirulent phenotypes. The mechanism of virulence of this pathogen is not well understood, particularly because it differs from other *Enterobacteriaceae* pathogens such as *Escherichia coli* and *Salmonella*. The capsule polysaccharide (CPS) of this pathogen is well recognized for contributing to the virulence of *K. pneumoniae*, but the exact mechanisms underlying its contribution are unclear. In this study, we demonstrated that CPS does not directly contribute to the host response; rather, it forms an external coat that blocks host recognition and prevents immune cells from binding to receptor proteins on *K. pneumoniae*, thus inhibiting phagocytosis, which makes it more challenging for the body to fight off infections. Understanding these mechanisms is vital for developing new treatments against *K. pneumoniae* infections, ultimately improving patient outcomes and public health.

## INTRODUCTION

*Klebsiella pneumoniae* is a human commensal and opportunistic pathogen that can cause several types of healthcare-associated infections (such as septicemia, pneumonia, urinary tract infections [UTI], and soft tissue infections [1]), especially among patients with a compromised immune system (2). Unlike classical *K. pneumoniae* (c*Kp*)*,* which often causes infections in immunocompromised individuals, hypervirulent *K. pneumoniae* (hv*Kp*) usually infects individuals in the community who are often healthy (3). Hv*Kp* is best described as a virulent pathogen (4); initial sequencing of hv*Kp* identified two large and highly similar plasmids pK2044 (5) and pLVPK (6), and loss of the plasmids resulted in significantly decreased virulence of hv*Kp* (7). To date, the best-characterized virulence factors that are known to confer the hypervirulence phenotype are encoded by genes present in these plasmids, including *iuc*, *peg-344*, *rmpA*, and *rmpA2*. Loss of either *rmpA* or *iucA* has a great impact on the virulence potential of *K. pneumonia*, with mean 16.7-fold and 9.6-fold lethality decrease, respectively (8). The inability to produce aerobactin, which is encoded by the *iuc* locus, also decreases *ex vivo* growth/survival of *K. pneumonia* in human ascites or serum. However, the abrogation of synthesis of other siderophores like enterobactin, yersiniabactin, and salmochelin has no significant impact on those aspects, suggesting that aerobactin is a critical virulence factor of hv*Kp* (9). PEG344, which may serve as an inner membrane transporter, also contributes to the survival and competition of *K. pneumonia* and is required for the full virulence in pulmonary challenge model (10). The hallmark clinical syndrome of hv*Kp* infection is a hepatic abscess in the absence of biliary tract disease, yet hv*Kp* can infect nearly every site of the body.

Among the known virulence factors of *K. pneumoniae*, CPS is considered the most important. The presence of a thick capsule at the cell surface protects *K. pneumoniae* from opsonization and phagocytosis by macrophages (11), neutrophils (12), epithelial cells (13), and dendritic cells (DCs) (14) by blocking the binding and internalization processes. CPS of *K. pneumoniae* also confers resistance to antimicrobial peptides. As a protective shield, CPS can limit the access of antimicrobial peptides to the bacterial cell. Moreover, sub-lethal concentrations of antimicrobial peptides in the airway induce *cps* gene expression, which, in turn, protects the bacteria against the action of antimicrobial polypeptides (15, 16). It was also reported that non-capsulated *K. pneumoniae* strains exhibited a fitness advantage by increasing biofilm formation and anti-serum-bactericidal-activity (anti-SBA) potential (17).

CPS also suppresses the early inflammatory response of the host by reducing IL-8 expression through inhibiting TLR2 and TLR4 signaling (18, 19) and NOD1-dependent pathways (20). *K. pneumoniae* CPS can also impair the maturation of DCs, resulting in reduced production of cytokines like IL-12 and TNF-α. These events allow the bacteria to evade host defenses and multiply *in vivo* more readily (14). However, some *in vitro* studies reported that CPS extracted from a K1 *K. pneumoniae* strain, NTUH-K2044, works as a factor that induces IL-1β secretion in an NLRP3-, ASC-, and caspase-1-dependent manner in macrophages (21). It is paradoxical that CPS was found to suppress and provoke the immune function of host cells in different studies; this discrepancy may be due to variation in experimental conditions in *in vivo* and *in vitro* studies.

In this work, we present comprehensive data on the functions of CPS in *K. pneumoniae* upon *in vivo* and *in vitro* infection. Our findings allow us to conclude that CPS confers the ability of the bacteria to survive *in vivo*. We also report the discovery of a new element involved in the phagocytosis process of *K. pneumoniae*, the scavenger receptor LOX-1. This study therefore provides new insights into the pathogenic mechanism of *K. pneumoniae* and corrects some conventional ideas regarding the role of CPS as a virulence factor.

## RESULTS

### Deficiency of CPS reduces the virulence of *Kp* of different capsule types to the same level

**Fig 1:**
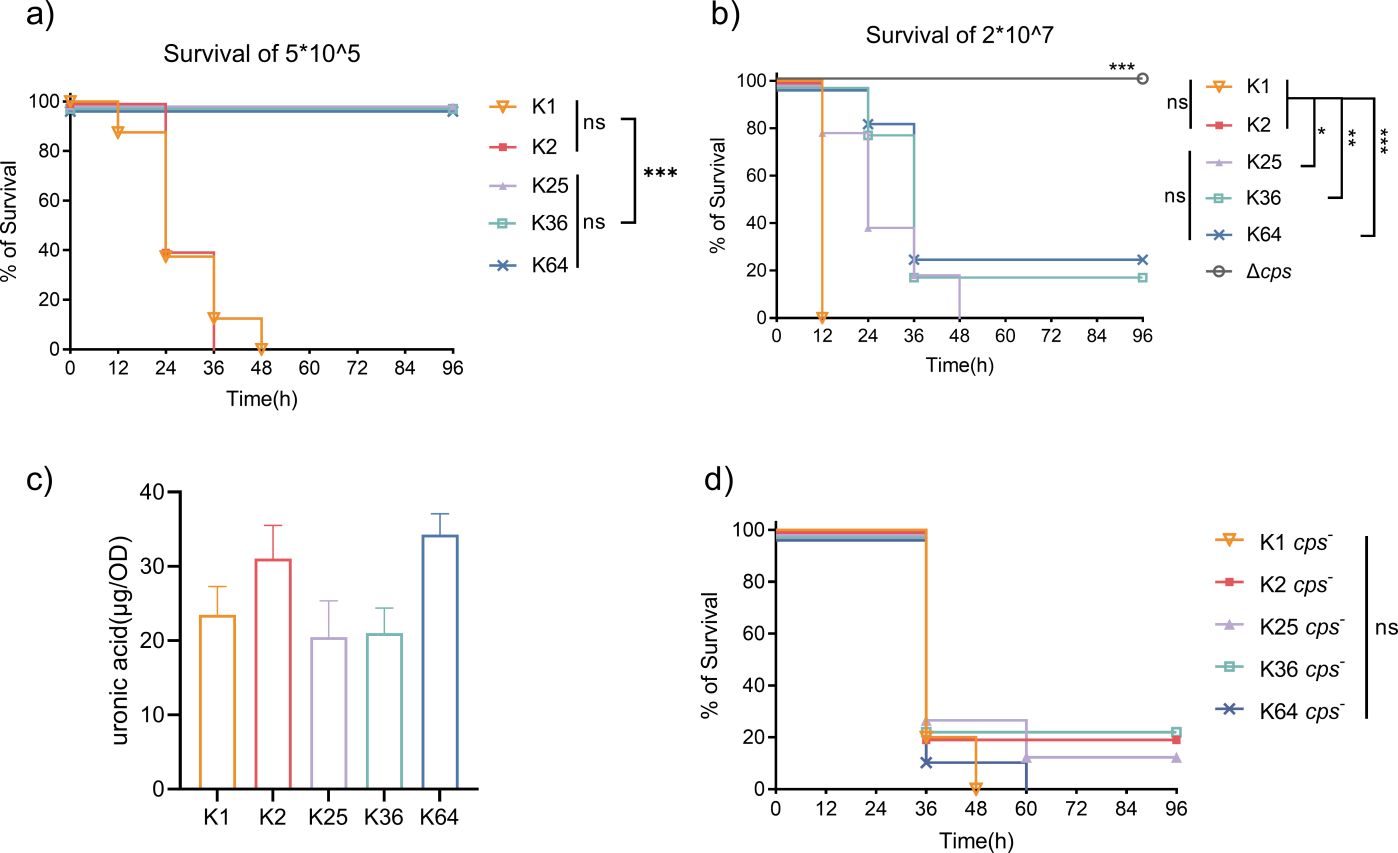
Assessment of the virulence level of *K. pneumoniae* isolates of different capsule types. Survival of C57BL/6 mice infected by i.p. injection of a dose of 5 × 10^5^ (a) or 2 × 10^7^ (b) of strains of K1, K2, K25, K36 and K64 capsule types was monitored. Strains were denoted by the following capsule types: K1 (KP1088PC), K2 (PM8PC), K25 (PM45), K36 (17ZR-22), and K64 (WZ1-2). *n* = 5. (c) The amount of CPS of K1, K2, K25, K36, and K64 capsule types was quantified by the uronic acid method. (d) The virulence level of CPS mutants of *K. pneumoniae* of different capsule types was determined by i.p. infection of C57BL/6 mice with 1 × 10^8^ CFU of each isolate. *n* = 6–7. Data were represented as mean ± SD, ns, not significant; *P < 0.05, **P < 0.01, ***P < 0.001.

To investigate how CPS contributes to the virulence potential of *K. pneumoniae* of different serotypes*,* clinical *K. pneumoniae* isolates were selected and tested for their virulence level using a mouse sepsis model. All virulence plasmids harbored by the test strains were cured to rule out the possibility that the virulence potential of the strains was determined by virulence factors or regulatory genes located in the plasmids. A total of five strains representing five different capsule types and serotypes were subjected to virulence assay, with results showing that these strains exhibited significantly different virulence levels (Fig. 1a and b). Mice infected with a dose of 5 × 10^5^ CFU of the isolates K25, K36, or K64 survived, but those infected by the same dose of isolates K1 and K2 died within 36 and 48 h, respectively (Fig. 1a). As shown in Figure 1b, infection with K1 and K2 at a high dose of 2 × 10^7^ caused 100% mortality in 12 h, and in 48 h with K25; however, infection with K36 and K64 was only associated with moderate mortality, with ~20% of mice remaining alive at the end of the 96 h of experiment. Although previous studies have proposed that capsule type defines the virulence level of *K. pneumoniae* (22), information on the genetic background of strains of different virulence levels is currently not available. Our data show that significant variation of virulence potential among isolates of different capsule types still exists upon removal of the virulence plasmids, indicating that chromosomal genetic elements play a key role in encoding the virulence of such strains.

We then evaluated the amount of CPS of isolates by uronic acid quantification to determine if the virulence level correlated with the CPS production in the test strains. As shown in Figure 1c, the amount of CPS produced did not correlate with the virulence potential of *K. pneumoniae*. Taking the K1 strain, which exhibited the highest level of virulence, as an example, this strain was found to produce only a moderate level of CPS among the tested strains. To test the degree of contribution of CPS to the variation of virulence potential of different isolates, isogenic mutants unable to produce CPS were generated by the two-plasmid CRISPR system pCasKP-pSGKP (23). These mutants were found to be avirulent in mice infected with bacterial doses of 2 × 10^7^ CFU when compared with their parental strains (Fig. 1b). When mice were inoculated with 1 × 10^8^ CFU of acapsular mutant of K1, K2, K25, K36, or K64, no significant difference in survival rate was detected, with about 20% survival rate being recorded in mice infected by each mutant (Fig. 1d). Based on this finding, we conclude that the virulence potential of *K. pneumoniae* is mainly determined by the capsule type rather than the amount of CPS, which was consistent with previous findings (22). These results revealed that inability to produce CPS results in a reduction of the virulence level of *K. pneumoniae* of different capsule types to the same level, regardless of the genetic background and carriage of other chromosomal virulence factors.

### CPS is essential for eliciting immunological responses upon *K. pneumoniae* infection and enhancing bacterial survival

**Fig 2:**
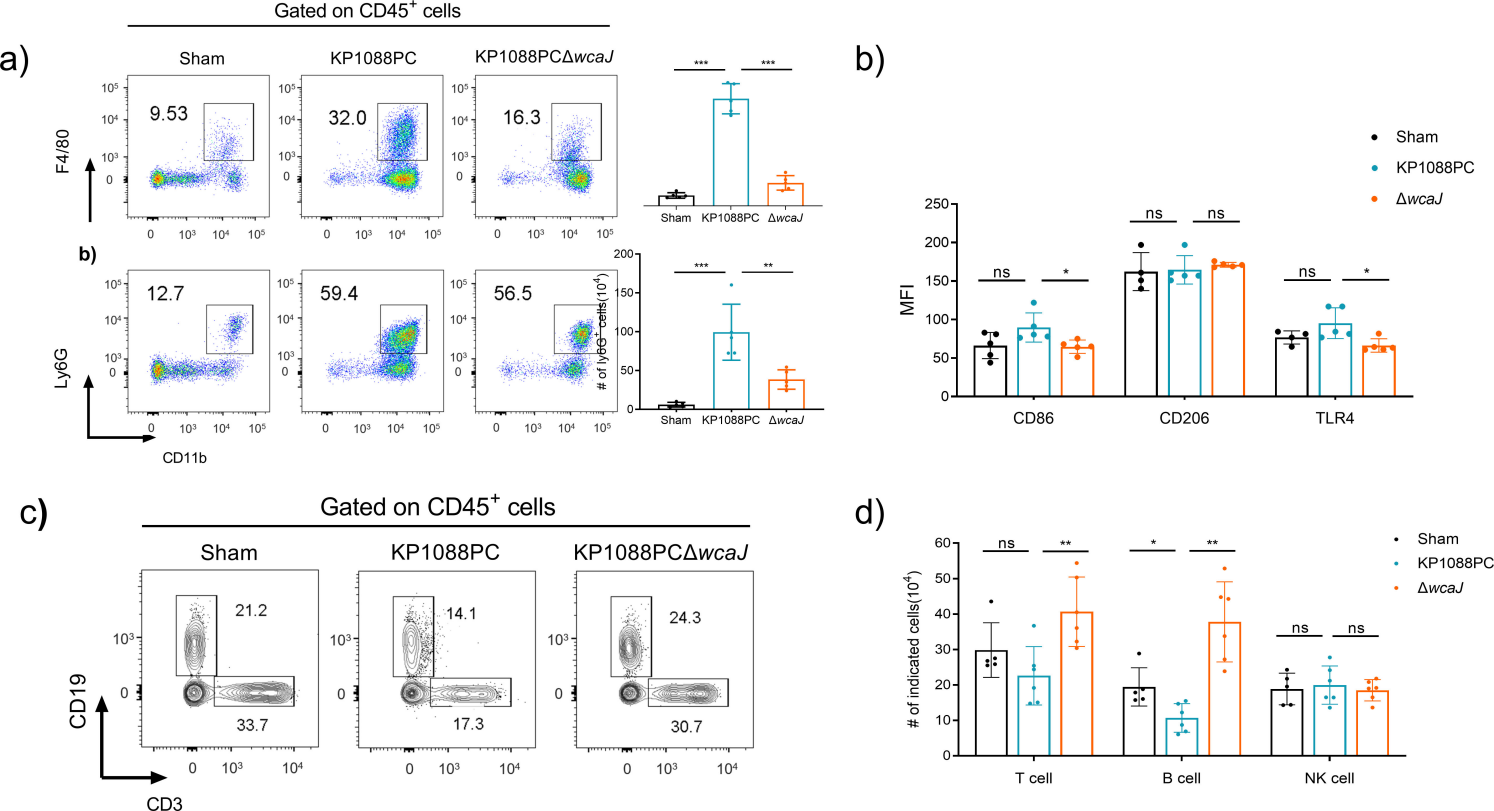
Immune landscape of mice upon infection by wild-type or CPS-deficient *K. pneumoniae* strain. Quantification of CD11b^+^F4/80^+^ cells and CD11b^+^Ly6G^+^ cells (a) on CD45^+^ cells in the lung tissues isolated from mice infected with KP1088PC or the CPS mutant strain by flow cytometry. (b) Quantification of mean fluorescence intensity (MFI) of CD86, CD206, and TLR4 on the surface of macrophages. Graphic representation (c) and quantification (d) of CD3^+^ and CD19^+^ cells in the lung tissues isolated from mice infected with KP1088PC or the CPS mutant strain by flow cytometry.

To further address the role of CPS in *K. pneumoniae* infection *in vivo*, mice were infected with a dose of K1 isolate KP1088PC that caused sepsis to develop rapidly. Briefly, the animals were inoculated with 5 × 10^5^ of the K1 wild-type (WT) or mutant strain, and were sacrificed at 12 hours postinfection (h.p.i) for analysis. Changes in the amount and types of immune cells in the lungs of mice were measured by flow cytometry to monitor the immune response of the host animals. Drastic increase in infiltration of neutrophils (CD11b^+^Ly6G^+^) and macrophages (CD11b^+^F4/80^+^) in the lungs was observed upon KP1088PC infection, when compared with the Sham group (Fig. 2a), which was consistent with our previous report of the immune profile induced by a K2 hv*Kp* strain 17ZR101 (24). Infection induced by the isogenic mutant led to a significantly lower level of infiltration of neutrophils and macrophages when compared with the parental strain, suggesting that the CPS-deficient strain triggered attenuated inflammatory responses in the host, and we hypothesize that this is attributed to the impaired ability of survival in the host due to the loss of CPS. Higher expression levels of CD86 were also found on macrophages in the lungs of KP1088PC-infected mice when compared with mutant-mediated infection and the Sham group (Fig. 2b). This finding indicates that *K. pneumoniae* infection skews macrophage differentiation toward the M1 subset (24), but this phenomenon was not seen in mice infected by the acapsular mutant. No significant change was detected in the expression of CD206, which is a marker for alternatively activated (anti-inflammatory M2) subsets of macrophages (Fig. 2b). Infection by the K1 isolate KP1088PC also induced lymphopenia in septicemic mice, which was marked by a deficiency of T cells and B cells, but such changes were not observable in mice infected by the acapsular strain (Fig. 2c and d). These results indicate that CPS plays a detrimental role in the immune response elicited by *K. pneumoniae*.

**Fig 3:**
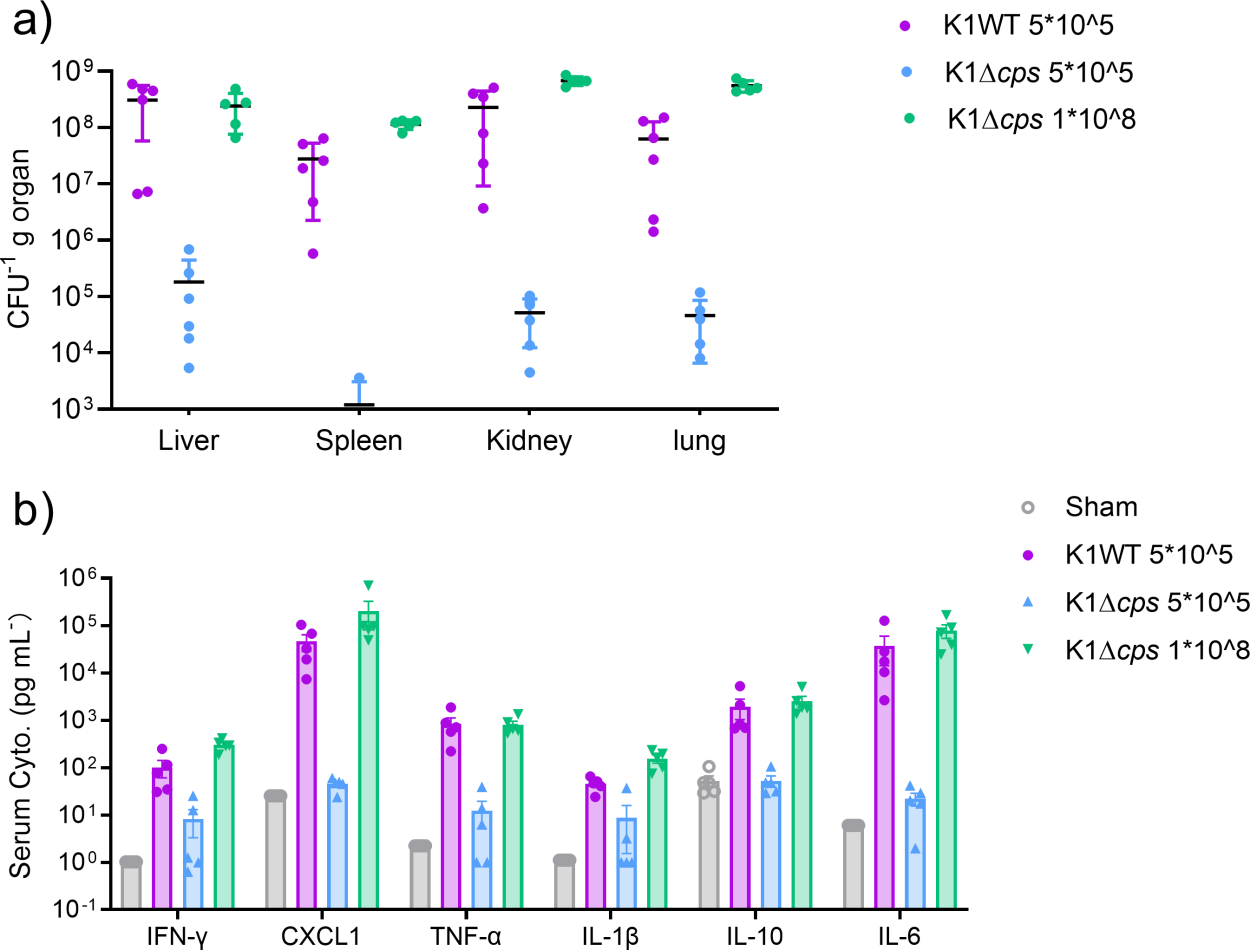
CPS is essential for the survival and immunogenicity of *Kp in vivo*. (a) Bacterial load in the liver, spleen, kidney, and lungs of mice 12 h after infection with K1 WT strain or the capsule mutant strain. (b) Profiling of levels of cytokines and chemokines in serum from Sham-treated mice and mice infected with K1 *K. pneumoniae* or Δ*cps* mutant strain.

To test our hypothesis and determine the invasiveness of the test strains, the number of viable bacteria in the organs of the infected mice, including the liver, spleen, kidney, and lungs, was determined by plate counting. The CFU count in acapsular-strain-infected mice was significantly lower than in wild-type-infected mice (Fig. 3a), indicating that CPS protects *K. pneumoniae* from being eradicated by the host immune response and enables better survival of *K. pneumoniae* in the host body. Moreover, analysis of the metabolite profile of serum collected from infected mice suggested that the WT *K. pneumoniae* infection provoked cytokine storm in the mice, with intensive release of cytokines and chemokines such as IFN-γ, TNF-α, IL-1β, IL-10, IL-6, and CXCL1 being observed (Fig. 3b). The abolishment of CPS production in *K. pneumoniae* diminished the ability to induce the release of such cytokines and chemokines in mice when inoculated with the same dose of WT strain (Fig. 3b).

As elimination of CPS almost abolished the changes in the profile of immune cells and stopped the elevation of cytokine levels caused by wild-type *K. pneumoniae* infection, we conclude that CPS is a major contributor of the immune response induced by *K. pneumoniae* infection. However, one question to be answered is whether the difference between the immunogenicity of the wild-type and CPS-deficient *K. pneumoniae* strain is attributed to the virulence factor CPS itself or the difference in the ability to survive within the host body. To answer this question, we inoculated mice with the Δ*cps* strain at a dose of 1 × 10^8^ CFU, which is able to induce sepsis. Notably, only with the higher challenging dose, the organ burden of Δ*cps-*infected mice reached the level of that recorded in the WT-infected mice; likewise, the level of the cytokines and chemokines triggered by a high dose of the Δ*cps* strain was comparable with that triggered by the wild-type strains (Fig. 3a and b). This observation indicates that *K. pneumoniae* lacking the CPS was still able to induce strong cytokine release when the number of bacteria inoculated into the test mice was high enough for it to survive and disseminate within the host body. These findings therefore suggest that CPS helps *K. pneumoniae* to escape from the host defense not through the elimination of host immune response but through the reduction of the host defense responses.

### CPS protects *K. pneumoniae* from phagocytosis but does not enhance intracellular survival

**Fig 4:**
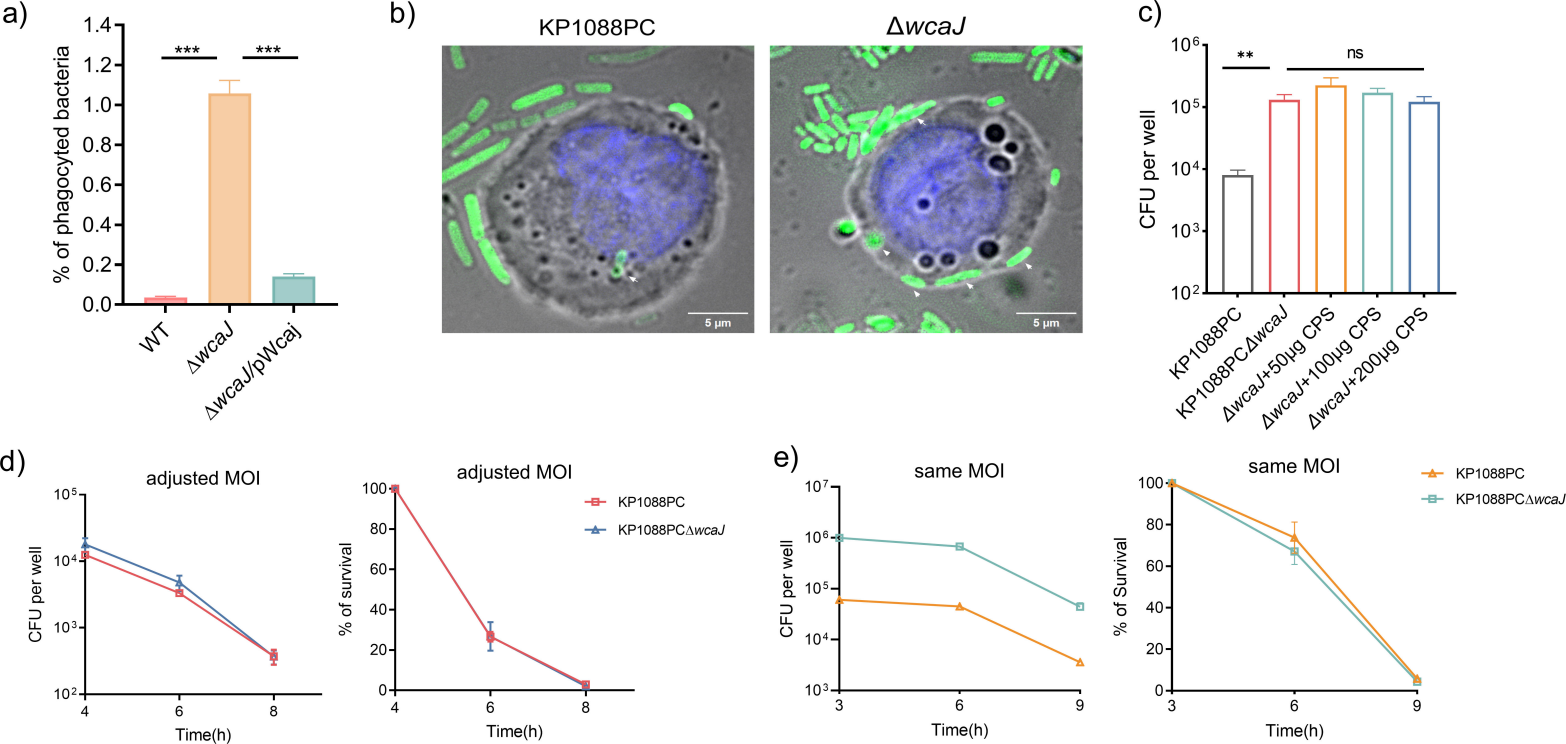
CPS affects the internalization but not the survival of *K. pneumoniae* in macrophage. (a) The percentage of phagocyted bacteria in mTHP-1 cells following infection with KP1088PC, the capsule mutant or the complement strain. (b) HIS-SIM image of mTHP-1 cells infected with the strain KP1088PC-*gfp* (left) or Δ*wcaJ-gfp* (right). Cell nuclei were stained by Hoechst 33258; the intracellular bacteria are indicated by white arrows. (c) Supplementation with purified CPS could not prevent the capsule mutant strain from being phagocyted. (d and e) Cessation of CPS synthesis or the number of engulfed bacteria has no impact on the intracellular survival of *K. pneumoniae*.

To investigate whether the role of CPS in *K. pneumoniae* pathogenesis is attributed to its ability to extend survival in the host, a macrophage infection model was adopted for further study on the interaction between the macrophage and bacterial cell *in vitro*. In this experiment, THP-1 cells were exposed to *K. pneumoniae* at a multiplicity of infection (MOI) of 25. A significant increase in the percentage of phagocyted bacteria was observed upon infection by the unencapsulated strain, when compared with infection by the parental strain, and complementation with the WcaJ-encoding plasmid was found to restore the capacity of *K. pneumoniae* to escape phagocytosis (Fig. 4a). Confocal microscopy studies further confirmed this finding by visualizing the intracellular bacteria using the *gfp*-expressing strain KP1088PC-*gfp* and Δ*wcaJ-gfp* (Fig. 4b). Notably, supplementation with purified CPS could not help the capsule-mutant strain escape from phagocytosis (Fig. 4c), indicating that cell-free CPS is unable to protect *K. pneumoniae* from being internalized.

We then monitored the fate of these intracellular strains. For the sake of comparison, we adjusted the MOI to obtain comparable numbers of intracellular bacteria at the beginning of the infection. However, there was no significant difference between the survival rate of the two strains (Fig. 4d). When inoculated with the same MOI, even though the number of internalized bacteria of the acapsular mutant was 10-fold more than that of the parental strain, the percentage of bacteria that survived in the macrophages did not change (Fig. 4d and e). These results show that the CPS of *K. pneumoniae* does not affect the bactericidal potential of macrophage, regardless of its effect on the capability of bacteria to escape from phagocytosis. Our data further illustrated that in the scenario of *in vivo* infection, the encapsulated strain was more resistant to phagocytosis and exhibited the same degree of viability as that of the CPS mutant strain, and that the WT strain tended to be resistant to clearance and accumulate extracellularly. Thereafter, prolonged survival and stimulation of the immune system can be one of the reasons why encapsulated bacteria elicited a stronger immune response.

### Involvement of the scavenger receptor LOX-1 in phagocytosis of *K. pneumoniae*

**Fig 5:**
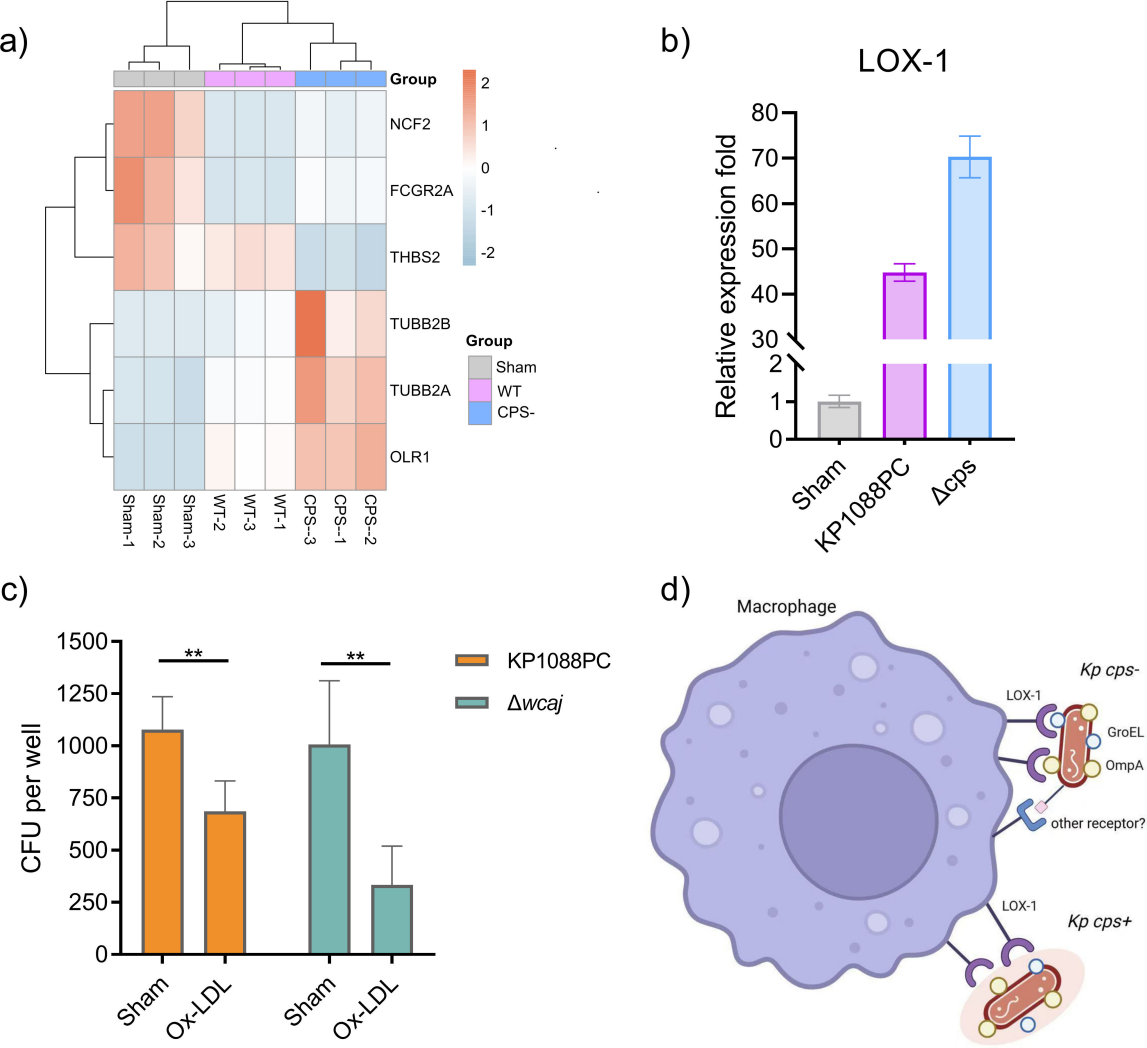
Involvement of LOX-1 receptor in *K. pneumonia* by macrophages. (a) Heatmap showing phagocytosis-related genes whose expression levels were altered significantly during *K. pneumoniae* infection. (b) mRNA expression level of LOX-1 in Sham-treated THP-1 cells or cells challenged with wild-type and CPS-deficient strain. (c) Blockage with the LOX-1 ligand Ox-LDL impaired the internalization of wild-type and unencapsulated *K. pneumoniae* strains. (d) Schematic representation of our hypothesis that CPS prevents *Kp* from being phagocyted by blocking the interaction of the scavenger receptor LOX-1 on macrophage with surface-associated GroEL and OmpA (generated by BioRender).

One of the questions that remains to be answered is how CPS prevents *K. pneumoniae* from being phagocyted. We performed RNA-seq on mTHP-1 cells infected with KP1088PC or the mutant strain unable to produce CPS. A total of 345 genes were found to express differentially in KP1088PC-treated and mutant-treated macrophages, of which six genes are involved in the process of phagocytosis (Fig. 5a). Among those genes was the *OLR1* gene that encodes a lectin-like oxidized low-density lipoprotein (LDL) receptor-1 (LOX-1) receptor protein, which is a scavenger receptor. We performed real-time quantitative PCR (qRT-PCR) to investigate the involvement of this receptor in the process of *Kp* infection. After stimulation with wild-type *K. pneumoniae*, the expression level of LOX-1 was significantly higher than that of the Sham control (Fig. 5b). Notably, the mRNA level of macrophage infected with the capsule mutant derivative was even higher than that of the parental strain-induced infection (Fig. 5b), suggesting that *K. pneumoniae* CPS affects the expression of LOX-1 on macrophages. Our result was consistent with a previous report that showed that *Escherichia coli* induced the expression of LOX-1 in mouse resident peritoneal macrophages (25).

As the scavenger receptor LOX-1 binds to ligands such as modified lipoproteins oxidized (Ox-) and acetylated (Ac-) LDL, we incubated mTHP-1 cells with Ox-LDL and tested the effects on *K. pneumoniae* internalization. As Figure 5c shows, Ox-LDL significantly impaired the phagocytosis of *K. pneumoniae* by macrophages. The reduction on internalization of wild-type *K. pneumoniae* by Ox-LDL was moderate but statistically significant; importantly, the percentage of inhibition was over 60% during infection by the CPS mutant (Fig. 5c). A previous study showed that LOX-1 recognizes and binds to the GroEL protein located at the surface of *E. coli* (25) and the outer membrane protein OmpA of *K. pneumoniae*, thereby enhancing adhesion of the macrophages to gram-negative bacteria and promoting phagocytosis of the pathogen (26). We thereby proposed that the scavenger receptor LOX-1 mediates phagocytosis of *K. pneumoniae* by macrophages probably by recognizing GroEL and OmpA, and that CPS blocks the recognition of *K. pneumoniae* by LOX-1 by providing a protective coat at the outermost layer of the bacteria, enabling the bacteria to evade engulfment by macrophages.

### *Kp* polarized M1 subtype in a CPS-independent way

**Fig 6:**
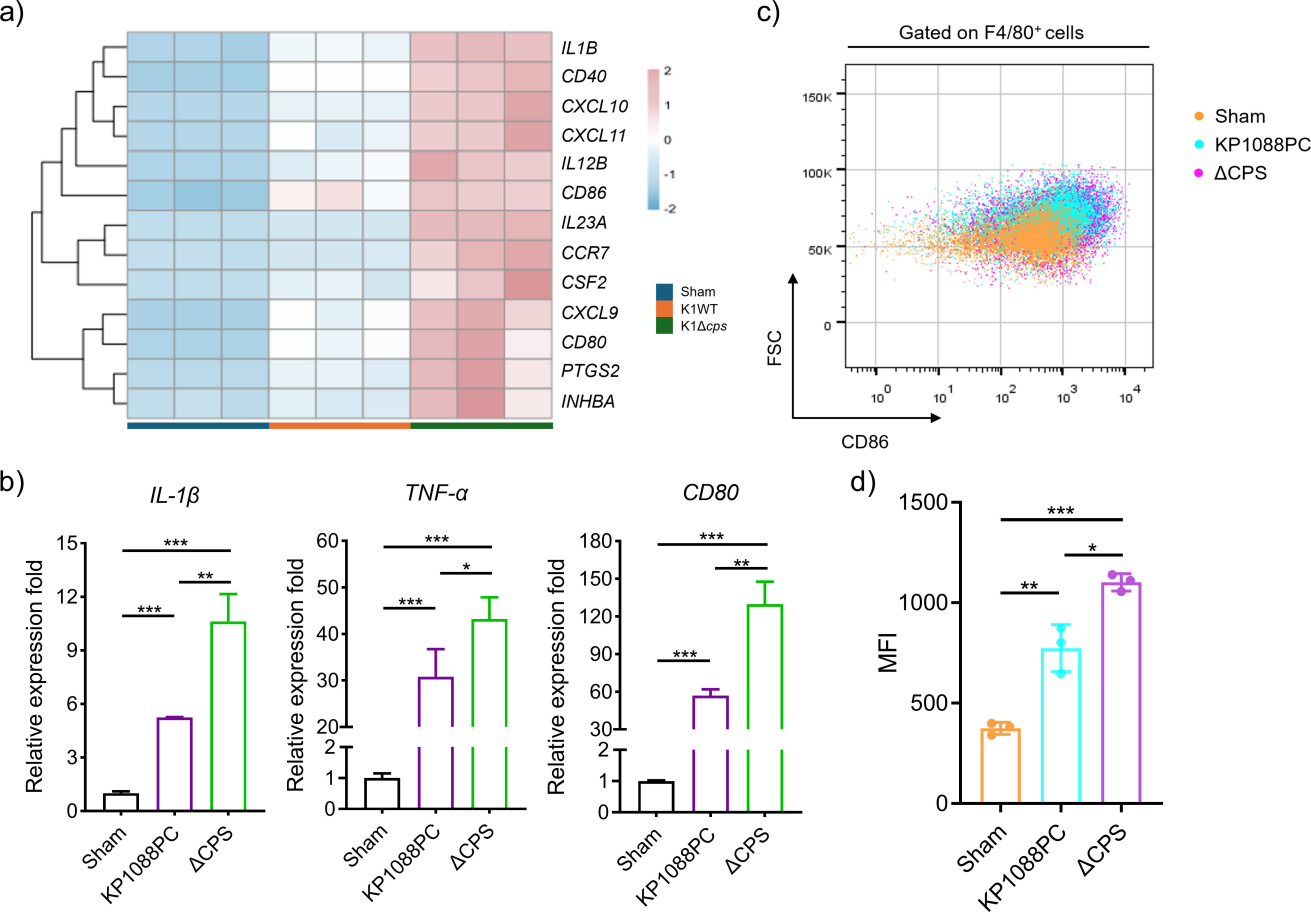
The induction of proinflammatory cytokines and M1 polarization by *Kp* are independent of CPS. (a) Heatmap of M1 polarization gene markers of mTHP-1 challenged with strain KP1088PC or its CPS-deficient mutant. (b) Expression level of IL-1β, TNF-α, and CD80 in Sham-treated THP-1 cell or cell challenged with wild-type and CPS-deficient strain. (c) Representative dot plots and (d) flow cytometry analysis showing the mean fluorescence intensity (MFI) of CD86 on RAW 264.7 cells in Sham condition or infected with wild-type or CPS mutant *Kp* strains.

Previously, we have reported that hv*Kp* infection triggers the recruitment of interstitial macrophages (IMs), which were found to express a higher level of CD80, suggesting that *Kp* infection can induce M1 polarization *in vivo* (24). Here, we observed this process only in WT-induced infection but not in infection induced by the isogenic CPS mutant (Fig. 2b). To further investigate the involvement of *Kp* CPS in the process of M1 polarization, we analyzed the transcriptome of monocyte-derived macrophage challenged with K1-*Kp* or the unencapsulated mutant strain. Notably, proinflammatory M1 polarization markers such as *Il23a*, *Socs3*, *Il1b*, and *Il1a* have a higher expression level in both WT- and mutant-infected macrophages compared with the Sham group, suggesting that CPS-deficient *Kp* was able to drive M1 polarization (Fig. 6a). Surprisingly, the transcriptional expression level of genes encoding proinflammatory cytokines IL-1β and TNF-α was significantly higher in the mutant-infected group compared with the WT-infected group, as well as the expression level of CD80, which is an M1 marker (Fig. 6b). For further investigation, fluorescence activated cell sorting (FACS) analysis was performed on RAW264.7 macrophage cells challenged with KP1088PC or the CPS-deficient strain. Consistently, *Kp* strains, whether producing CPS or not, were able to induce M1 polarization of macrophages, with higher mean fluorescence intensity of CD86 detected on macrophages infected with the CPS mutant strain (Fig. 6c and d). These observations indicated that *Kp* induces M1 polarization in a CPS-independent way, and it is further convincing that the unencapsulated *Kp* strain not only retain its immunogenicity but also trigger high M1 polarization due to its high phagocytosis rate. The failure of induction of M1 polarization and cytokine storm by CPS knockout strain in our *in vivo* model can be attributed to its impaired survival capability within the host. As we observe a higher frequency of M1 macrophage during CPS-deficient strain infection, we then speculated that internalized bacteria were responsible for screwing macrophage toward M1 subtype. These results showed that *Kp* can modulate the production level of CPS to manipulate macrophage polarization state.

## DISCUSSION

In this work, we conducted a comprehensive study on the role of the virulence factor CPS in the pathogenesis of *K. pneumoniae*. CPS has been considered a prime virulence factor of *K. pneumoniae*, the functions of which have been a subject of research for decades. More than 79 capsular serotypes have been reported to date, with significant differences in virulence level being observed among different capsular types, of which K1 and K2 strains were found to be particularly virulent in a mouse peritonitis model (27, 28). However, as virulence plasmids were frequently detected in isolates that express the capsule antigen K1 (29), it cannot be concluded that the virulence potential is defined by the capsule type of the strains, especially previous reports showed that virulence plasmids carried various virulence factors such as *iuc* (coding for aerobactin), *peg344* (a metabolic transporter), and *rmpA* and *rmpA2* (regulators of capsule production) (3). A study by Huang et al. reported that, among a total of 81 clinical isolates tested, all K1, K2, K16, and K20 strains exhibited the hypervirulent phenotype, which produced a cutoff value of 50% mortality within 7 d of infection in a mouse intraperitoneal (i.p.) model (22). The researchers also replaced the *cps* gene cluster in a K2 isolate with other strains that expressed different capsule antigens, and the virulence level of hybrid strains was consistent with the CPS donor strain. However, only one strain was tested using such method, and no data of the low-virulence strains, which had acquired a *cps* gene cluster from a high-virulence strain, were presented. Here, we constructed isogenic Δ*cps* mutants of five representative strains of five different capsule types, and tested the virulence trait of the parental and mutant strains. We found that isolates of different capsule types still exhibited distinct virulence potential when the virulence plasmid was removed from the bacterial cell, and that the virulence level of the test strains did not correlate with the level of CPS production. Importantly, abolishment of CPS synthesis function was found to bring the virulence level of various *K. pneumoniae* strains down to the same level, which was nearly avirulent. Based on these data, we are confident to draw a conclusion that capsule type is the determining factor for the expression of the virulence phenotype in *K. pneumoniae*.

Our research group has previously reported that the high mortality of hv*Kp* infection was attributed to the induction of cytokine storm associated with the strong and overactive immune responses triggered in the host during the infection process (24). In this study, we found that deletion of CPS-encoding genes impaired the ability of *K. pneumoniae* to induce immune response and to survive *in vivo*. However, it is still not clear whether the reduced immunogenicity of the capsule-deficient strain is because (i) CPS is responsible for activating certain inflammatory signaling pathways, or (ii) the mutant strain can be more readily eradicated by the host when compared with the wild-type strain. We then challenged the mice with a high dose of Δ*cps* strain, and the data showed that, while the bacterial load of unencapsulated strain reached the same level as that of the wild-type strain, the level of IFN-γ, TNF-α, IL-12, IL-1β, IL-10, and IL-6 in the serum was found to increase significantly when compared with the Sham-treated group. This finding indicates that *K. pneumoniae* strains that are unable to produce CPS also trigger the onset of a cytokine storm when inoculated at a high dose. We therefore conclude that (i) the virulence factor CPS is not the only cellular determinant of *K. pneumoniae* that can activate inflammatory responses and is not necessary for the induction of the release of cytokines, and that (ii) CPS contributes to the prolonged survival of *K. pneumoniae*, thus resulting in a stronger immune response.

We were then eager to investigate the interaction between *K. pneumoniae* and the host using *in vitro* macrophage infection model. We observed resistance to phagocytosis in encapsulated strain, which is consistent with findings of previous studies (30). Although CPS prevents *K. pneumoniae* from being internalized, it is indispensable for intracellular survival of the bacteria, regardless of the MOI. This observation can further explain why encapsulated strains can survive better during the infection process. Despite exhibiting the same intracellular survival rate, *K. pneumoniae* strain producing CPS is more resistant to internalization and therefore accumulates at a higher rate extracellularly.

We identified a scavenger receptor, namely LOX-1, whose expression level was upregulated during *K. pneumoniae* infection. It was reported that the scavenger receptor LOX-1 mediates the adherence of *E. coli* to macrophages by recognizing the GroEL protein (25), and that deletion of the gene that encoded this receptor suppressed the induction of IL-1β, IL-18, and TNF-α by *K. pneumoniae* outer-membrane vesicles (OMVs) (31). qRT-PCR results confirmed the upregulation of LOX-1 upon infection, which was about 40-fold and 70-fold in wild-type and unencapsulated-strain-infected macrophage, respectively, when compared with the Sham-treated group. To further study the functional role of LOX-1, mTHP-1 cells were blocked with a ligand of LOX-1, Ox-LDL. The blockage impaired the phagocytosis of both wild-type and capsule-deficient *K. pneumoniae,* and we observed a comparatively more functional inhibition of internalization of the capsule-deficient strain. These results suggest that the LOX-1 receptor mediates the internalization of *K. pneumoniae,* and CPS prevents this interaction possibly by providing a protective layer on the surface of bacteria, likely preventing exposure of proteins like GroEL and OmpA. This is one of the reasons why CPS helps *K. pneumoniae* escape phagocytosis.

It was interesting that the transcriptional analysis of the WT- or mutated-*Kp*-infected macrophage showed CPS does not play a large role in triggering the immune response of the host cell in our *in vitro* model. The *cps* mutant did not display any loss of ability to induce inflammatory response upon infection and even triggered a higher level of IL-1β and TNF-α in macrophages. Furthermore, the *cps* mutant was also able to induce M1 polarization. These findings may seem inconsistent with our *in vivo* data. However, the difference in the condition of infection must be taken into consideration. In the *in vitro* model, we only focus on the impact of intracellular bacteria on one cell type of the host, which is the monocyte-derived macrophage, while it is much more complicated in the scenario of *in vivo* study. The fact observed in our *in vitro* model that loss of CPS does not attenuate the ability of *Kp* to induce inflammatory response helps explain the formation of cytokine storm in mice challenged with a high dose of CPS-deficient strain.

It is worth noting that *K. pneumoniae* can regulate the expression of CPS to achieve a different biological status, and it has been reported that *Klebsiella* downregulates the expression of *cps* when residing within the *Klebsiella*-containing vacuole (KCV) when being phagocyted (30). As our research mainly focuses on the perspectives of host side, future efforts could be devoted to how the pathogen responds to the immune clearance, e.g., transcriptomic and proteomics analysis of intracellular bacteria or bacteria residing in the organs of the host.

To summarize, our research indicates that the capsule type is the major chromosomal determinant of the virulence level of *K. pneumoniae* strains, and that the ability of *K. pneumoniae* to establish systemic infection and induce the immune response is impaired when CPS production has ceased. We explain the discrepancy between the observation in *in vitro* and *in vivo* studies by illustrating that the ability of CPS to help *K. pneumoniae* escape phagocytosis contributes to higher survival fitness within the host body and the potential to induce a stronger cytokine storm. Further investigation is required for delineating the underlying mechanisms by which different K types define the virulence level of the organism and how CPS serves as a protective shell of *K. pneumoniae* during host–pathogen interactions.

## MATERIALS AND METHODS

### Bacterial strains, plasmids, and culture conditions

The *Klebsiella* strains used in this study were recovered from clinical samples of patients admitted to different hospitals located in mainland China and the Hong Kong Special Administrative Region. Bacterial strains and plasmids used in this study are listed in Table 1. *E. coli* and *K. pneumoniae* strains were grown in Luria-Bertani (LB) broth at 37°C. Bacterial density was estimated by measuring the absorbance at 600 nm. Accurate CFU was determined for each experiment by plating an aliquot on LB agar plates. If required, antibiotic was added at the following concentrations: kanamycin 50 µg/mL, apramycin 50 µg/mL, spectinomycin 50 µg/mL for selection.

**TABLE 1:** Strains and plasmids used in this study

Strain	Description	Reference or source
DH5α	*Escherichia coli* DH5α	NEB
KP1088PC	*Klebsiella pneumoniae* strain, K1	(32)
PM8PC	*K. pneumoniae* strain, K2	(32)
PM45	*K. pneumoniae* strain, K25	This study
17ZR-22	*K. pneumoniae* strain, K36	This study
WZ1-2	*K. pneumoniae* strain, K64	(32)
KP1088PCΔ*wcaJ*	KP1088PC derivative; Δ*wcaJ*	This study
PM8PCΔ*wcaJ*	PM8PC derivative; Δ*wcaJ*	This study
PM45Δ*wcaJ*	PM45 derivative; Δ*wcaJ*	This study
17ZR-22Δ*wcaJ*	17ZR-22 derivative; Δ*wcaJ*	This study
WZ1-2Δ*wcaJ*	WZ1-2 derivative; Δ*wcaJ*	This study
KP1088PCΔ*wcaJ/*pWcaJ	KP1088PCΔ*wcaJ* carrying pBAD-WcaJ, complement strain, Kan^R^	This study
KP1088PC-*gfp*	KP1088PC derivative carrying pSGKP-spe-GFP, Spe^R^	This study
KP1088PCΔ*wcaJ-gfp*	KP1088PCΔ*wcaJ* derivative carrying pSGP-spe-GFP, Spe^R^	This study
pCasKp-apr	Temperature-sensitive plasmid carrying Cas9 gene and lambda red recombination genes, Apr^R^	(23)
pSGKp-spe	Plasmid contains the sgRNA with the synthetic J23119 promoter and the *sacB* gene for plasmid curing, Spe^R^	(23)
pBAD18	Plasmid containing the arabinose PBAD promoter, Kan^R^	ATCC
pSGKp-spe-GFP	pSGKP-spe derivative expressing GFP, Spe^R^	This study

### Genome editing and complementary plasmid construction

Genome editing was performed following the steps described by Wang et al. (23). For complementary plasmid construction, the *cps* gene of K1 strain was amplified from *K. pneumoniae* strains KP1088 and was then cloned into the vector pBAD18; the recombinant plasmids were transformed into the *cps* mutant strains.

### Mouse infection model

Six- to eight-week-old animals of both sexes were used in this study. WT C57BL/6 mice were bred in-house. All mice were housed and bred under specific pathogen-free conditions. All experiments were performed using sex- and age-matched controls, were approved by The Hong Kong Polytechnic University Shenzhen Research Institute, and followed the guidelines of the Institutional Laboratory Animal Research Unit. Mouse bacteremia model was established in C57BL/6 mice (6–8 wk) to test the virulence level of *K. pneumoniae* strains. Log-phase bacteria were collected and resuspended in phosphate-buffered saline (PBS) to the desired concentration in a final volume of 200 µL for intraperitoneal inoculations. C57BL/6 mice (6–8 wk) were infected intraperitoneally, and their survival was recorded for 96 h postinfection. The Kaplan-Meier survival curves were generated using GraphPad Prism 7.00.

### Tissue preparation and flow cytometry

Cells collected from different tissues of the test animals were subjected to flow cytometry analysis as described previously (33). Single cell suspension of spleen was obtained by mashing the organ through a 70 µm cell strainer and collecting the tissue in a tube containing RPMI 1640 medium supplemented with 5% fetal bovine serum (FBS). To prepare lung cells, lung tissues were excised and incubated in Hanks' balanced salt solution (HBSS) containing 1× HEPES and collagenase type I. The tissue fragments were forced through a 70 µm strainer as described above. Red blood cells were lysed with ACK lysing buffer. Dead cells were excluded from the analysis by propidium iodide (Sigma, P4170-1G) staining in all flow cytometry experiments. Fluorescently labeled mAbs CD45 (Cat# 103106), CD11b (Cat# 101235), CD206 (Cat# 141703), CD86 (Cat# 105029), Ly6G (Cat# 127615), CD3 (Cat# 100235), CD19 (Cat# 115529), CD4 (Cat# 100437), CD8 (Cat# 100751), NK1.1 (Cat# 156513), F4/80 (Cat# 123113), TLR4 (Cat# 145405), and appropriate isotype controls were obtained from BioLegend. Flow cytometric analyses were performed using a BD FACSCelesta flow cytometer (BD Bioscience). The acquired data were analyzed by the FlowJo software (Version 10.0.7, Treestar, Palo Alto, CA, USA).

### Cytokine and chemokine assessment

Blood was collected from the test mice by orbital bleeding and was subjected to centrifugation at 10,000 × *g* for 25 min at 4°C to obtain the serum. The serum was stored immediately at −80°C for further analysis. The serum level of IFN-γ, TNF-α, IL-1β, IL-10, IL-6, and CXCL1 was determined using LEGENDplex Mouse Anti-Virus Response Panel (BioLegend, 740621) according to the instructions of the manufacturer.

### Cell culture

Human monocytes THP-1 (ATCC, TIB-202) were grown in RPMI 1640 tissue culture medium supplemented with 10% FBS, 1% nonessential amino acid (Gibco), and 1% penicillin-streptomycin at 37°C in a humidified 5% CO_2_ atmosphere. THP-1 cells were differentiated into macrophages by phorbol 12-myristate 13-acetate (PMA) treatment (150 ng/mL for 48 h).

### Adhesion and invasion assay

THP-1 cells were seeded into a 24-well plate for differentiation at a density of 4 × 10^5^ cells per well 48 h before the experiment. Bacteria at mid-log phase were harvested and washed with PBS, then aliquoted into each well to achieve an MOI = 20 in FBS-free RPMI 1640. For adherence assay, the plates were centrifuged for 5 min at 200 × *g* to synchronize the infection process. The plates were incubated at 37°C in a humidified 5% CO_2_ atmosphere. After 30 min of contact, the cells were washed three times with PBS and lysed by adding 500 µL of 0.4% Triton X-100. The adherence rate was calculated by recording the proportion of the inoculum that adhered to the wells of the plate. To perform invasion assays, the cells were washed with PBS after 2 h of infection, and the medium containing 300 µg/mL amikacin was added to the wells to eradicate extracellular bacteria. After a second incubation for 2 h, the cells were lysed with 0.4% Triton X-100, and the number of intracellular bacteria was determined by plating serial dilutions of the lysate onto LB agar and incubating for 12 h at 37°C. The invasion rate was the proportion of the inoculum that was internalized.

### RNA extraction and real-time quantitative PCR assay

RNA was extracted from THP-1 cells in TRIzol reagent (15596026, Thermo Fisher Scientific), followed by chloroform extraction and isopropanol precipitation, and dissolved in 44 µL of RNase-free water. THP-1 cells were seeded into a 6-well plate at a density of 2 × 10^6^ per well and were allowed for differentiation for 48 h. Cells were infected with *Kp* at an MOI = 5 for 2 h, followed by incubation in RPMI medium containing 300 µg/mL amikacin for 6 h. DNA was removed from the extracted RNA samples using TURBO DNA-free Kit, and the reverse transcription was carried out using SuperScript III First-Strand Synthesis SuperMix kit (11752050, Thermo Fisher Scientific). Real-time quantitative PCR was performed by using a QuantStudio 5 Real-Time PCR System following the manufacturer’s instructions. Primers are listed in Table 2. The relative expression level of the samples was determined by the comparative threshold cycle (ΔΔCT) method in triplicate, using the glyceraldehyde-3-phosphate dehydrogenase gene (*GAPDH*) for normalization.

**TABLE 2:** Primers for qPCR in this study

Gene	Strand	Sequence 5′−3′
*GAPDH*	Forward	GAGTCAACGGATTTGGTCGT
	Reverse	GACAAGCTTCCCGTTCTCAG
*IL1β*	Forward	TCCAGGGACAGGATATGGAG
	Reverse	TCTTTCAACACGCAGGACAG
*TNFα*	Forward	CCGAGGCAGTCAGATCATCTT
	Reverse	AGCTGCCCCTCAGCTTGA
*CD80*	Forward	CTCACTTCTGTTCAGGTGTTATCCA
	Reverse	TCCTTTTGCCAGTAGATGCGA
*LOX1*	Forward	GCACAGCTGATCTGGACTTCAT
	Reverse	CCCCATCCAGAATGGAAAACT

### Transcriptomic analysis

RNA samples were sent to Novogene (Hong Kong) Company Limited for sequencing. Quality control, mRNA purification, and library preparation were performed by the company. Sequencing read alignment was performed by using HISAT2. Count-aligned reads and quantification were calculated based on exon regions using FeatureCounts. Differentially expressed genes were identified by DESeq2 analysis. Gene Set Enrichment Analysis was performed for gene ontology enrichment analysis.

### Ox-LDL blocking assay

THP-1 cells were seeded into a 96-well plate at a density of 4 × 10^4^ cells per well, followed by incubation with 100 µg/mL of Ox-LDL for 1 h. The bacterial culture was then added, and the intracellular CFU was counted.

### *In vitro* detection of polarization

RAW264.7 macrophage cells were cultured in Dulbecco's modified Eagle's medium (DMEM) supplemented with 10% (vol/vol) heat-inactivated FBS, 1% penicillin and streptomycin. The cells were seeded into a 6-well tissue culture plate at a density of 1.25 × 10^6^ cells/well and were allowed to incubate for around 24 h prior to infection. Log-phase *Kp* strains were inoculated at an MOI = 20, and after 2 h of infection, the macrophages were washed with PBS and further incubated in DMEM containing 300 µg/mL gentamycin for 22 h. Cells were collected, washed once, and stained with PE-Cy7 anti-F4/80 and APC-Cy7 anti-CD86 in FACS buffer according to the manufacturer’s instructions. Propidium iodide was added at a final concentration of 1 µg/mL before flow cytometry analysis.

## Data Availability

The raw RNA-seq data have been deposited in NCBI database under the BioProject accession number PRJNA1195673 (SAMN45224892 to SAMN45224900).
